# Blueberry Anthocyanins Extract Attenuates Acrylamide-Induced Oxidative Stress and Neuroinflammation in Rats

**DOI:** 10.1155/2022/7340881

**Published:** 2022-05-23

**Authors:** Zizhuang Fang, Yinghua Luo, Chen Ma, Li Dong, Fang Chen

**Affiliations:** College of Food Science and Nutritional Engineering, National Engineering Research Center for Fruits and Vegetables Processing, Key Laboratory of Storage and Processing of Fruits and Vegetables, Ministry of Agriculture, Engineering Research Centre for Fruits and Vegetables Processing, Ministry of Education, China Agricultural University, Beijing 100083, China

## Abstract

Acrylamide (AA) is a widespread environmental and dietary-derived neurotoxin, which can induce oxidative stress and associated inflammation in the brain. Anthocyanins widely occur as natural antioxidant and anti-inflammatory phytochemicals. Herein, the protective effects of blueberry anthocyanins extract (BAE) against AA-induced neurotoxicity were investigated in rats. The rats were pretreated with BAE (175 mg/kg body weight/day) by oral gavage for the first 7 days, followed by the co-administration of BAE and AA (35 mg/kg body weight/day) by oral gavage for the next 12 days. Results showed that BAE significantly decreased the malondialdehyde (MDA) production, and increased glutathione (GSH) and antioxidant enzyme levels; and it also suppressed microglial activation, astrocytic reaction, and pro-inflammatory cytokine expressions. Furthermore, BAE elevated the extracellular signal-related kinase (ERK)/cAMP response elements binding protein (CREB)/brain-derived neurotrophic factor (BDNF) pathway, and relieved the accumulation of amyloid beta (A*β*) 1-42 and 1-40 after AA exposure. Consequently, AA-induced neuronal necrosis and downregulation of synaptosomal-associated protein 25 (SNAP-25) were attenuated by BAE in the hippocampus and cerebral cortex. In conclusion, BAE can exert a protective function on neurons and synapses against AA-induced oxidative stress and neuroinflammation.

## 1. Introduction

Industrial activities produce a large amount of hazardous chemicals, which can affect human health and even cause serious physical disorders. Acrylamide (AA), a synthesis vinyl monomer, is widely applied in wastewater treatment, ore processing, and cosmetics manufacturing [1]. In addition, AA can also be produced during the thermal processing of carbohydrate-rich foods, such as potato chips, bread, and coffee [1]. It has been reported that AA exhibits neurotoxicity, reproductive toxicity, genotoxicity, and potential carcinogenicity *in vivo* and *in vitro* [1–3]. In particular, AA-induced neurotoxic symptoms have been demonstrated in exposed workers, including ataxia, weight loss, sensation disturbances, and locomotor defects [1]. Moreover, a recent prospective cohort study of 2534 aged people suggested that AA intake may also be related to the cognitive decay of Chinese elderly men [4]. Therefore, the neurotoxic risk of AA exposure has raised considerable concern in the world.

Oxidative stress triggered by AA is generally believed to be the main cause of its neurotoxicity [5]. AA can induce the accumulation of reactive oxygen species (ROS) and lipid peroxidation, accompanied by high levels of malondialdehyde (MDA); it can also increase the consumption of glutathione (GSH) and inhibit the expressions of antioxidant enzymes, such as superoxide dismutase (SOD), catalase (CAT), and glutathione peroxidase (GSH-Px) [5]. As a result, the disruption of redox-homeostasis by AA can damage intracellular proteins, lipids, and DNA in the brain, which triggers a variety of downstream reactions and even leads to neuronal death and synaptic failure [6]. In previous studies, AA-induced oxidative stress was suggested to promote inflammatory response in the brain, during which activated microglia and reactive astrocytes overproduce pro-inflammatory cytokines such as interleukin-1*β* (IL-1*β*), interleukin-6 (IL-6), and tumor necrosis factor-alpha (TNF-*α*), thus aggravating the toxicity of AA to neurons and synapses [7–9]. Furthermore, excessive ROS and pro-inflammatory molecules can reduce the levels of brain-derived neurotrophic factor (BDNF), which is a crucial neurotrophin and plays a regulatory role in brain development, synaptic plasticity, and neurogenesis [10]. In rats, AA administration downregulated the expressions of BDNF in the hippocampus and cerebral cortex by blocking the extracellular signal-related kinase (ERK)/cAMP response elements binding protein (CREB) pathway, thereby suppressing its multi-neuroprotective effects [11, 12]. Consequently, the decline of BDNF expressions would in turn exacerbate oxidative stress and neuroinflammation, and eventually form a vicious circle [10].

Several phytochemicals have been demonstrated to play a protective role against AA neurotoxicity owing to their strong antioxidant ability, such as curcumin [11], punicalagin [13], ellagic acid [14], and rutin [15]. As a class of widespread flavonoids in fruits and vegetables, anthocyanins are easily available in people's daily diet (50 mg/day recommended by China Nutrition Society) and are well known for their dramatic free radical scavenging and anti-inflammatory efficacies [16, 17]. Additionally, with the capacity to cross the blood-brain barrier (BBB) in a bilitranslocase-dependent manner, anthocyanins have presented the ability to inhibit ROS generation as well as microglia- and astrocytes-mediated neuroinflammation in the brain, thus preventing neurodegeneration and cognitive deficits in lipopolysaccharide (LPS)-injected, d-galactose-induced, or APP/PSEN1 transgenic rodent models [18–20]. Based on these benefits, the interventional effects of food-derived anthocyanins against AA neurotoxicity would be worthy of exploration. Our previous studies have indicated that blueberries are rich in anthocyanins [21]; and blueberry anthocyanins extract (BAE) can prevent AA-induced ROS production by reducing the epoxidation of AA to its metabolite glycidamide with higher oxidative toxicity in the lung, liver, and kidney [21–23]. However, the actions of BAE on the neurotoxicity caused by AA remain unclear to date.

In the present study, we investigated for the first time whether BAE can exert neuroprotective functions on neurons and synapses against AA-induced oxidative stress in the hippocampus and cortex of rats. Moreover, the impacts of BAE on neuroinflammation and the ERK/CREB/BDNF signaling pathway were also studied. Besides, accumulating evidence has indicated that the deposition of amyloid beta (A*β*), which is considered to be synaptotoxic and a histopathologic hallmark of Alzheimer's disease (AD), can also be induced by oxidative stress and neuroinflammation [24, 25]. Nevertheless, whether AA exposure can accelerate A*β* accumulation has not been reported yet. These enlightened us to further explore the toxicology of AA for A*β* accumulation and the detoxification potential of BAE in this study.

## 2. Materials and Methods

### 2.1. Materials and Chemicals

The blueberry (*Vaccinium uliginosum*) anthocyanins extract (BAE) was obtained from the Daxinganling Lingonberry Organic Foodstuffs Co., Ltd. (Daxinganling, China). Identification and quantification of anthocyanins in BAE using HPLC/ESI/MS were described in our previous study [21, 26]. The total content of anthocyanins in BAE was 257.6 ± 2.3 mg/g, of which cyanidin-3-glucoside was as high as 90%, and the rest were cyanidin-3,5-diglucoside and peonidin-3-glucoside [21]. BAE was dissolved to the concentration of 1.75% in saline solution before use. AA (purity >99%) was obtained from Sigma-Aldrich (St. Louis, MO, USA), and was dissolved to the concentration of 0.7% in saline solution before use.

### 2.2. Animal Experiment

**Figure 1:**
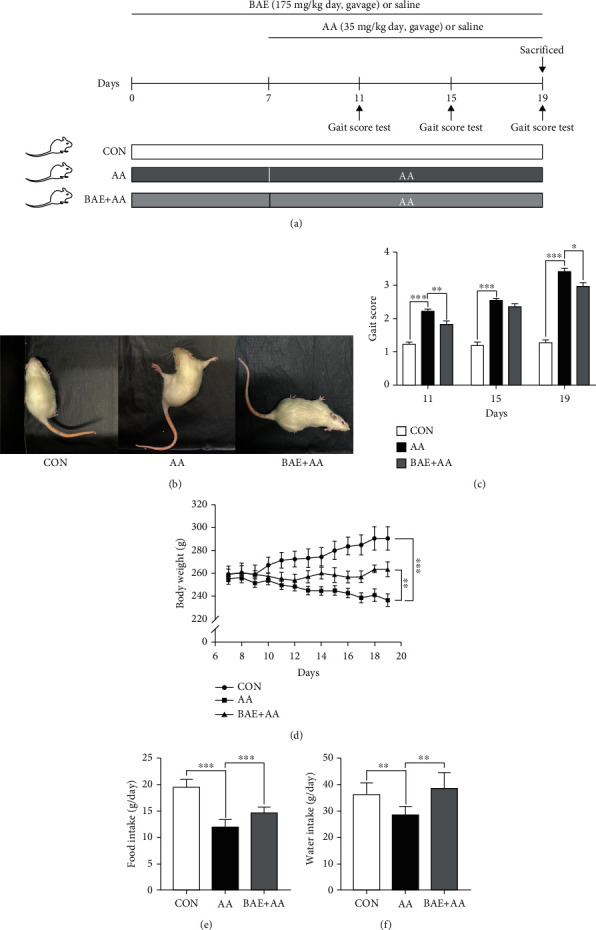
Blueberry anthocyanins extract (BAE) attenuated gait abnormalities and weight loss in acrylamide (AA)-treated rats. (a) Experimental procedure of rats grouping and treatment. (b) Representative behavior performance at the end of experiment. (c) Gait scores on days 11, 15, and 19. (d) Body weight, (e) food intake, and (f) water intake since day 7. Data are mean ± SEM (*n* =8). ∗*p* < 0.05, ∗∗*p* < 0.01, ∗∗∗*p* < 0.001. CON: saline gavage; AA: AA gavage; BAE+AA: BAE pretreatment followed by AA gavage.

Eight-week-old female Sprague Dawley (SD) rats were provided by Vital River Laboratory Animal Technology Co. Ltd (Beijing, China), which were kept under specific-pathogen-free (SPF) facility (22.0 ± 1.0°C, 55 ± 5% humidity, 12 h/12 h light/dark cycle) with libitum access to standard diet and drinking water. All animal experiments were approved by China Agricultural University Laboratory Animal Welfare and Animal Experimental Ethical Committee (Beijing, China) (No. AW92901202-4-1). As shown in Figure 1(a), after a week of acclimatization to the laboratory conditions, the rats were randomly divided into 3 groups (*n* =8 per group) and representing (i) the control group (CON) received 19-day oral gavage of saline (0.9% NaCl) with 10 mL/kg body weight/day; (ii) the AA group (AA) received 12-day oral gavage of AA with 35 mg/kg body weight/day after 7-day gavage of saline; (iii) the BAE+AA group (BAE+AA) received 7-day oral gavage of BAE with 175 mg/kg body weight/day, which was followed by co-administration of BAE and AA by oral gavage for the next 12 days. Female rats were chosen in the experiment based on their significantly higher bioavailability of AA than males, which indicated that females could be more susceptible to AA exposure [27]. The dose of BAE and AA was selected based on our previous studies [21, 22].

At the end of this experiment, the rats were sacrificed after sodium pentobarbital anesthesia. The blood samples were collected by eyeball enucleation, and centrifuged at 7,000x*g* at 4°C for 5 min after incubating at room temperature to obtain the serum samples. The brain tissues were collected, half of which were freshly immersed in 4% paraformaldehyde for histological analysis, and the other half were dissected into cerebral cortex and hippocampus, immediately frozen in liquid nitrogen, and stored at -80°C for molecular analysis.

### 2.3. Gait Score Test

Gait scores were investigated on days 11, 15, and 19 by a previously described method [11] (Figure 1(a)). The rats were observed individually for 3 min in a clean ground, and quantified with gait scores ranging from 1 to 4. Score 1 represented normal, unaffected gaits; score 2 represented slightly abnormal gaits characterized by mild foot splay and hind limb weakness during ambulation; score 3 represented moderately abnormal gaits characterized by obvious foot splay and hind limb weakness during ambulation; and score 4 represented severely abnormal gaits characterized by severe foot splay with dragging hind limbs as well as incapacity to support body weight and retreat. The behavioral assessment was conducted by 5 blinded observers who were trained and not participating in the study, and then the average score of each rat was calculated.

### 2.4. Histological Analysis

Collected brain samples were fixed for 48 hours with 4% paraformaldehyde. Following paraffin embedding, the brain tissue blocks were sectioned at a thickness of 4 *μ*m, and subjected to hematoxylin and eosin staining (H&E staining) and Congo red staining. The histological sections were observed and photographed at 100 or 200x magnification under an optical microscope (Carl Zeiss, Oberkochen, Germany). Necrotic and normal neurons in the hippocampus dentate gyrus region (DG), cornu ammonis region 1 (CA1), cornu ammonis region 3 (CA3), and cerebral cortex were counted at least four sections per rat to provide a mean value.

### 2.5. Immunohistochemistry

After deparaffinization with xylene and rehydration with sodium citrate antigen repair solution (Solarbio, Beijing, China), the brain sections were blocked with 5% bovine serum albumin (BSA) and washed with phosphate-buffered saline (PBS). The tissue sections were incubated with ionized calcium binding adaptor molecule-1 (IBA-1) (sc-32725, Santa Cruz Biotechnology, Santa Cruz, CA, USA), glial fibrillary acidic protein (GFAP) (sc-33673, Santa Cruz Biotechnology) antibodies overnight at 4°C. Following washing with PBS, the brain sections were used a peroxidase-conjugated secondary antibody (Huaxingbio, Beijing, China) for 2 hours at room temperature, and visualized with diaminobenzidine (DAB) chromogenic substrate (Zsbio, Beijing, China). The positive cell areas of IBA-1 and GFAP were measured with ImageJ software (National Institutes of Health, Bethesda, MD, USA), while the positive cell numbers were counted at least four sections per rat to provide a mean value.

### 2.6. ELISA Assay

The expressions of SOD, CAT, and GSH-Px and levels of MDA, GSH, IL-1*β*, IL-6, TNF-*α*, A*β*1–42, and A*β*1–40 in the hippocampus, cerebral cortex tissue lysates, or serum were examined by ELISA kit (Mlbio, Shanghai, China). The total protein contents were qualified with a BCA protein assay kit (Solarbio).

### 2.7. Western Blot

Hippocampus and cerebral cortex tissues were homogenized with RIPA buffer (Beyotime, Shanghai, China) supplemented with protease and phosphatase inhibitors (Solarbio). After centrifugation at 12,000x*g*, 4°C for 15 min, the supernatant was used to quantify the protein concentration by BCA assay. Then, the proteins were separated by 10% sodium dodecyl sulfate poly acrylamide (SDS-PAGE) gels, and transferred onto polyvinylidene fluoride (PVDF) membranes (Millipore, Bedford, MA, USA) by electrophoresis. After blocked with 5% nonfat milk for 2 h under room temperature, the blots were incubated with primary antibodies to synaptosomal-associated protein 25 (SNAP-25) (sc-65508, Santa Cruz Biotechnology), postsynaptic density protein 95 (PSD-95) (sc-32290, Santa Cruz Biotechnology), BDNF (ab108319, Abcam, Cambridge, MA, USA), CREB (#9197, Cell Signaling Technology, Danvers, MA, USA), phospho-CREB (p-CREB) (Ser133) (AF3189, Affinity, Cincinnati, OH, USA), ERK (BF8004, Affinity), phospho-ERK (Thr202/Try204) (p-ERK) (#4370, Cell Signaling Technology), and *β*-actin (ab8227, Abcam) overnight at 4°C. The next day, secondary antibodies conjugated with horseradish peroxidase (Huaxingbio) were probed for 2 h at room temperature. After treated with SuperSignal chemiluminescent substrate (Huaxingbio), the blots were developed and analyzed using enhanced chemiluminescence system (Tanon, Shanghai, China). The density levels were quantified by ImageJ software.

### 2.8. Statistical Analysis

All data were expressed as the mean ± SEM. GraphPad Prism Version 7.0 (GraphPad Software, San Diego, CA, USA) was performed to compare all groups using one-way ANOVA followed by Tukey's post hoc test. *p* < 0.05 was considered statistically significant.

## 3. Results

### 3.1. BAE Attenuated AA-Induced Gait Abnormalities and Weight Loss in Rats

In the experiment, the rats were pretreated with BAE (175 mg/kg body weight/day) by oral gavage for the first 7 days, followed by the co-administration of BAE and AA (35 mg/kg body weight/day) by oral gavage for the next 12 days (Figures 1(a)). Behaviorally, AA-treated rats exhibited slightly abnormal gaits on day 11 (*p* < 0.001), and developed characteristic symptoms of AA toxicity on day 19, including foot splay, twisting of hind limbs, and difficulty in ambulation with dramatically higher gait scores (*p* < 0.001) (Figures 1(b) and 1(c)). Fortunately, BAE supplementation attenuated the gait abnormalities and decreased the gait scores of rats after AA exposure (*p* < 0.05 on days 11 and 19, *p* > 0.05 on day 15) (Figures 1(b) and 1(c)). Moreover, BAE remarkably improved the AA-triggered body weight loss (*p* < 0.01), as well as food (*p* < 0.001) and water intake (*p* < 0.01) reduction (Figures 1(d)–1(f)).

### 3.2. BAE Ameliorated AA-Induced Neuronal and Synaptic Damages in Rats

**Figure 2:**
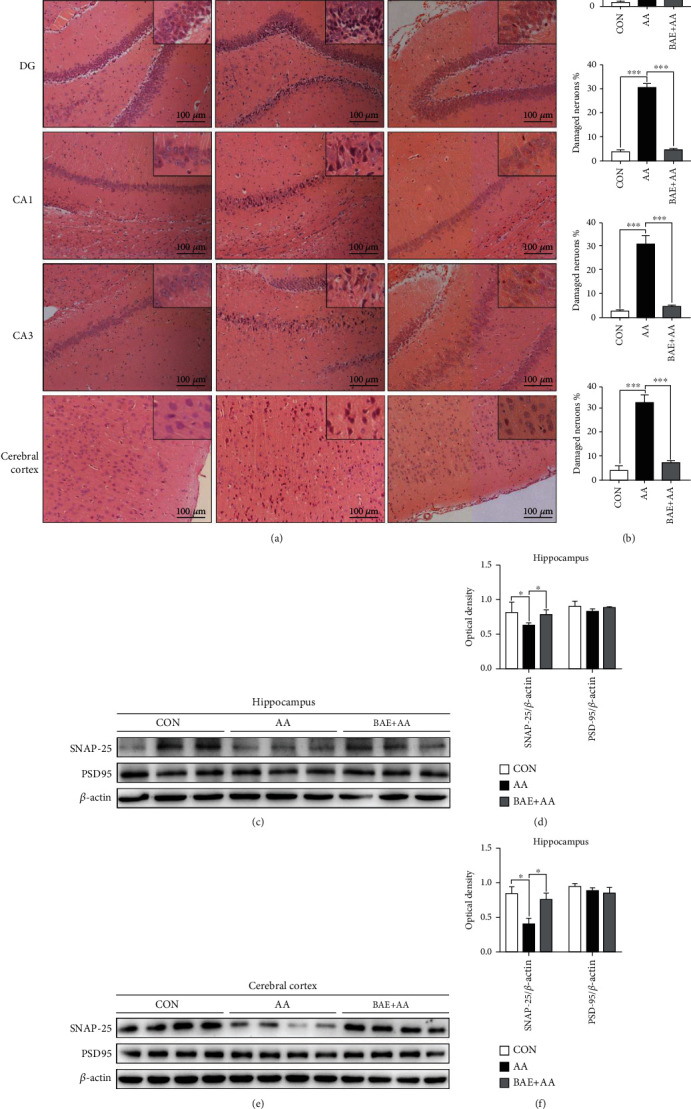
Blueberry anthocyanins extract (BAE) ameliorated neuronal and synaptic damages in acrylamide (AA)-treated rats. (a) Representative H&E staining in the hippocampus dentate gyrus region (DG), cornu ammonis region 1 (CA1), cornu ammonis region 3 (CA3), and cerebral cortex (100×, scale bar =100 *μ*m). (b) Percentage of damaged neurons (*n* =4). Western blot of synaptosomal-associated protein 25 (SNAP-25) and postsynaptic density protein 95 (PSD-95) in the (c) hippocampus and (d) cerebral cortex (*n* =3 or 4). Relative expression of SNAP-25/*β*-actin and PSD-95/*β*-actin in the (e) hippocampus and (f) cerebral cortex (*n* =3 or 4). Data are mean ± SEM. ∗*p* < 0.05, ∗∗∗*p* < 0.001. CON: saline gavage; AA: AA gavage; BAE+AA: BAE pretreatment followed by AA gavage.

Neuronal and synaptic impairments in the hippocampus and cerebral cortex result in a wide range of brain disorders [28]. In the present study, the neuronal histopathological damages were examined by H&E staining, and the number of damaged neurons was counted. As shown in Figure 2(a), in the CON group, neurons in the hippocampus DG, CA1, CA3 regions and cerebral cortex were normal with well-preserved cytoplasm, prominent nucleus, and nucleolus. Meanwhile, AA induced profound neuronal degeneration with concentrated nuclei, darkly stained basophilic chromatin, and disordered arrangement, whereas the above neuronal damages were significantly ameliorated by BAE treatment (*p* < 0.05) (Figures 2(a) and 2(b)).

Furthermore, in order to evaluate whether BAE can exert synaptic protection against AA, the pre- and postsynaptic proteins, SNAP-25 and PSD-95, were measured by Western blot in rat hippocampus and cortex. AA administration caused a significant reduction in SNAP-25 protein levels in the hippocampus and cortex (*p* < 0.05), whereas BAE reversed them to levels close to the CON group (*p* < 0.05) (Figures 2(c)–2(f)). However, there was no obvious difference in the PSD-95 expressions among the CON, AA, and BAE+AA groups, possibly because the damage of AA occurred on the presynaptic functions (such as axonal transport) rather than postsynaptic functions [5] (Figures 2(c)–2(f)).

### 3.3. BAE Alleviated AA-Induced Oxidative Stress in Rats

**Figure 3:**
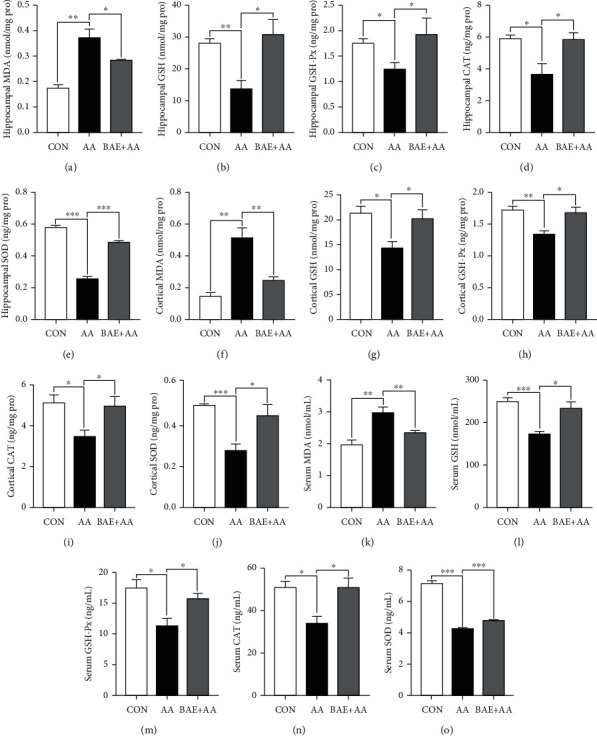
Blueberry anthocyanins extract (BAE) alleviated oxidative stress in acrylamide (AA)-treated rats. Hippocampal, cortical, and serum levels of ((a), (f), (k)) malondialdehyde (MDA), ((b), (g), (l)) glutathione (GSH), ((c), (h), (m)) glutathione peroxidase (GSH-Px), ((d), (i), (n)) catalase (CAT), and ((e), (j), (o)) superoxide dismutase (SOD). Data are mean ± SEM (*n* =4). ∗*p* < 0.05, ∗∗*p* < 0.01, ∗∗∗*p* < 0.001. CON: saline gavage; AA: AA gavage; BAE+AA: BAE pretreatment followed by AA gavage.

It is believed that oxidative stress in the brain is the main cause of AA neurotoxicity [5]. Therefore, the effect of BAE on AA-induced redox imbalance was investigated. As a biomarker of lipid peroxidation, a significantly higher MDA concentration in the hippocampus, cortex, and serum (*p* < 0.01) was observed in the AA group (Figures 3(a), 3(f), and 3(k)), while the main components of cellular antioxidant systems, GSH, GSH-Px, CAT, and SOD, decreased simultaneously (*p* < 0.05) (Figures 3(b)–3(e), 3(g)–3(j), and 3(l)–3(o)). However, BAE supplementation effectively alleviated AA-induced increase of MDA (*p* < 0.05), and reduction of GSH (*p* < 0.05), GSH-Px (*p* < 0.05 in the cortex and serum, *p* > 0.05 in the hippocampus), CAT (*p* < 0.05), and SOD levels (*p* < 0.05) in the hippocampus, cortex, and serum (Figures 3(a)–3(o)), suggesting that BAE treatment can drastically prevent the AA-induced oxidative stress.

### 3.4. BAE Reduced AA-Induced Microglial Activation and Astrocytic Reaction in Rats

**Figure 4:**
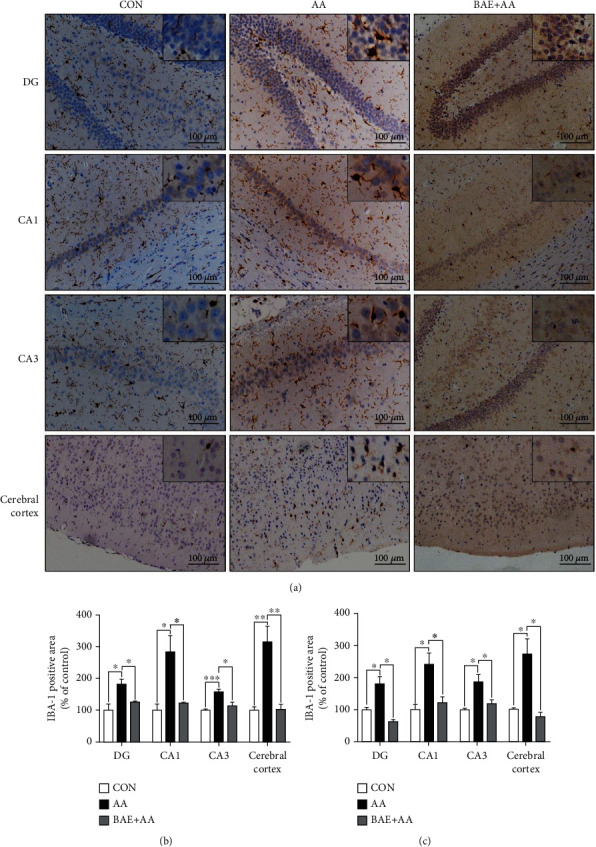
Blueberry anthocyanins extract (BAE) reduced microglial activation in acrylamide (AA)-treated rats. (a) Representative immunohistochemical staining of ionized calcium binding adaptor molecule-1 (IBA-1) in the hippocampus dentate gyrus region (DG), cornu ammonis region 1 (CA1), cornu ammonis region 3 (CA3), and cerebral cortex (100×, scale bar =100 *μ*m). (b) Relative area and (c) relative numbers of IBA-1 positive cell. Data are mean ± SEM (*n* =4). ∗*p* < 0.05, ∗∗*p* < 0.01, ∗∗∗*p* < 0.001. CON: saline gavage; AA: AA gavage; BAE+AA: BAE pretreatment followed by AA gavage.

**Figure 5:**
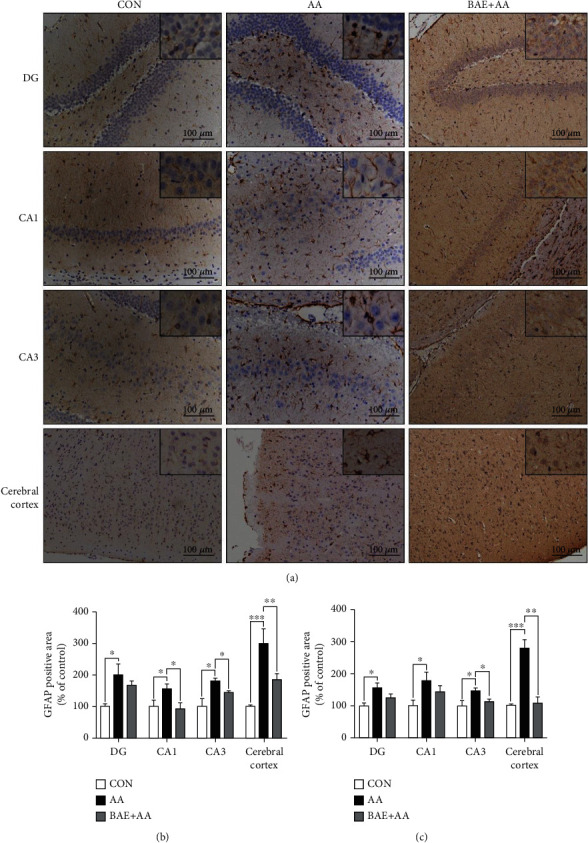
Blueberry anthocyanins extract (BAE) mitigated astrocytic reaction in acrylamide (AA)-treated rats. (a) Representative immunohistochemical staining of glial fibrillary acidic protein (GFAP) in the hippocampus dentate gyrus region (DG), cornu ammonis region 1 (CA1), cornu ammonis region 3 (CA3), and cerebral cortex (100×, scale bar =100 *μ*m). (b) Relative area and (c) relative numbers of GFAP-positive cell. Data are mean ± SEM (*n* =4). ∗*p* < 0.05, ∗∗*p* < 0.01, ∗∗∗*p* < 0.001. CON: saline gavage; AA: AA gavage; BAE+AA: BAE pretreatment followed by AA gavage.

Inappropriate microglial activation and astrocytic reaction can be promoted by oxidative damage in the brain, leading to further toxicity to neurons and synapses [7]. In the present study, the expressions of IBA-1 and GFAP, as specific markers of microglia and astrocytes, were measured by immunohistochemical staining to indicate the microglial activation and astrocytic reaction in the rat brain. In the CON group, resting microglia exhibited normal small cell bodies with thin processes (Figure 4(a)), and astrocytes were stained brown with numerous thin and branched processes (Figure 5(a)). However, in the AA group, microglia were highly activated characterized by ameboid phenotype with hypertrophied cell bodies and thickened processes (Figure 4(a)), while astrocytes were reacted showing stained dark brown cell bodies and hypertrophic processes (Figure 5(a)). Meanwhile, the population of microglia and astrocytes was significantly increased after AA exposure (*p* < 0.05) (Figures 4(b) and 4(c) and 5(b) and 5(c)). With the supplementation of BAE, the microglial activation (*p* < 0.05) and astrocytic reaction (*p* < 0.05 in the CA1, CA3, and cortex, *p* > 0.05 in the DG) induced by AA were relieved in the hippocampus and cortex (Figures 4(a)–4(c) and 5(a)–5(c)).

### 3.5. BAE Decreased AA-Induced Pro-Inflammatory Factor Overproduction in Rats

**Figure 6:**
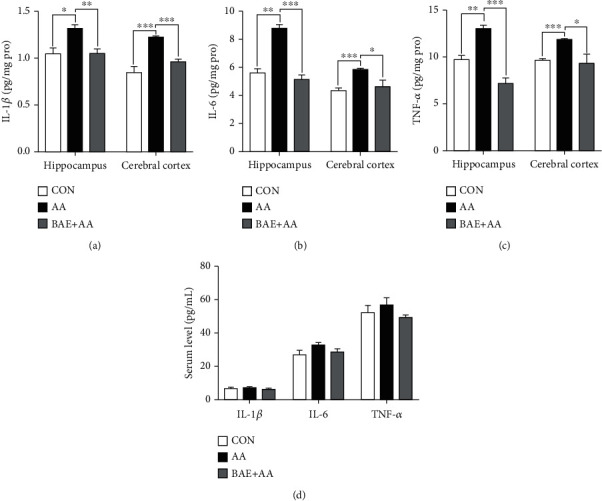
Blueberry anthocyanins extract (BAE) decreased pro-inflammatory factors in acrylamide (AA)-treated rats. (a) Interleukin-1*β* (IL-1*β*), (b) interleukin-6 (IL-6), and (c) tumor necrosis factor-alpha (TNF-*α*) levels in the hippocampus and cortex. (d) Serum IL-1*β*, IL-6, and TNF-*α* levels. Data are mean ± SEM (*n* =4). ∗*p* < 0.05, ∗∗*p* < 0.01, ∗∗∗*p* < 0.001. CON: saline gavage; AA: AA gavage; BAE+AA: BAE pretreatment followed by AA gavage.

Overproduction of pro-inflammatory factors including IL-1*β*, IL-6, and TNF-*α* by microglia and astrocytes is considered to be a critical event in the neuroinflammation [7]. Compared with the CON group, AA induced marked expressions of IL-1*β*, IL-6, and TNF-*α* in both the hippocampus and cortex (*p* < 0.05), which were significantly reversed by BAE administration (*p* < 0.05) (Figures 6(a)–6(c)). In agreement with the results in Figures 4 and 5, these findings showed that BAE supplementation decreased AA-induced neuroinflammation in rat hippocampus and cortex. Nevertheless, in serum, no appreciable difference in the expressions of these pro-inflammatory factors was observed after AA treatment (Figure 6(d)), indicating that AA-induced inflammation in the brain may precede the one in peripheral circulation [29].

### 3.6. BAE Improved AA-Induced Reduction of ERK/CREB/BDNF Signaling Pathway in Rats

**Figure 7:**
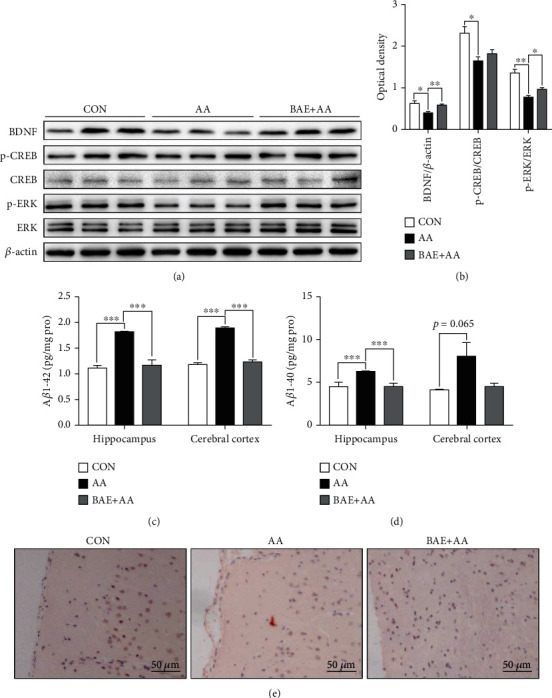
Blueberry anthocyanins extract (BAE) improved the extracellular signal-related kinase (ERK)/cAMP response elements binding protein (CREB)/brain-derived neurotrophic factor (BDNF) pathway and reduced amyloid beta (A*β*) accumulation in acrylamide (AA)-treated rats. (a) Western blot of BDNF, p-CREB, CREB, p-ERK, and ERK in the hippocampus (*n* =3). (b) Relative expression of BDNF/*β*-actin, p-CREB/CREB, and p-ERK/ERK (*n* =3). (c) A*β*1-42 and (d) A*β*1-40 levels in the hippocampus and cortex (*n* =4). (e) Representative Congo red staining in the cortex (200×, scale bar =50 *μ*m). Data are mean ± SEM. ∗*p* < 0.05, ∗∗*p* < 0.01, ∗∗∗*p* < 0.001. CON: saline gavage; AA: AA gavage; BAE+AA: BAE pretreatment followed by AA gavage.

The ERK/CREB pathway plays a crucial role in regulating BDNF, which is an important neurotrophic factor involved in maintaining neuronal survival and synaptogenesis [10]. When its Ser133 is phosphorylated by kinase ERK, CREB can facilitate the gene expressions of BDNF [11]. To further evaluate the protective effect of BAE on neurons and synapses, we assessed the protein expressions of ERK/CREB/BDNF signaling pathway in rat hippocampus. Results showed that AA treatment caused a substantial reduction in BDNF protein levels (*p* < 0.05), and blocked the phosphorylation of ERK (*p* < 0.01) and CREB (*p* < 0.05) in the hippocampus compared with the CON group (Figures 7(a) and 7(b)). Notably, BAE supplementation efficiently normalized BDNF expressions (*p* < 0.01) and p-ERK/ERK ratio (*p* < 0.05), and showed a tendency to increase in p-CREB/CREB ratio (*p* > 0.05) (Figures 7(a) and 7(b)). These findings indicated that BAE attenuated AA-induced reduction of BDNF by improving ERK/CREB signaling pathway in the hippocampus of rats.

### 3.7. BAE Inhibited AA-Induced Accumulation of A*β* in Rats

A*β* is the main component of amyloid plaques in the brains of AD patients and animal models, of which A*β*1–42 and A*β*1–40 are the most abundant forms [24, 25]. It is demonstrated that A*β* accumulation can be induced by oxidative stress and neuroinflammation [24, 25]. However, whether AA exposure can accelerate A*β* deposition remains unclear. In the present study, we found for the first time that AA induced an enrichment of A*β*1–42 (*p* < 0.001 in the hippocampus and cortex) and A*β*1–40 (*p* < 0.001 in the hippocampus, *p* = 0.065 in the cortex) (Figures 7(c) and 7(d)), whereas BAE inhibited the expressions of A*β*1–42 (*p* < 0.001 in the hippocampus and cortex) and A*β*1–40 (*p* < 0.001 in the hippocampus, *p* > 0.05 in the cortex) after AA exposure (Figures 7(c) and 7(d)). As shown in Figure 7(e), the Congo red-stained amyloid plaques were observed in the cortex of AA-treated rats, while none of them in the CON or BAE+AA group. These results suggested the inhibitory effect of BAE on AA-induced A*β* expression and deposition.

## 4. Discussion

AA is known as a neurotoxic environmental contaminant, which is also commonly found in a variety of heated food products owing to the Maillard reaction [1]. Nowadays, the health risks of AA exposure and the possible interventions against AA have become public concerns. In the present study, AA-induced oxidative stress in the brain was believed to be one of the main causes for its neurotoxicity. AA-triggered lipid peroxidation with high levels of MDA, and the imbalance of the antioxidant systems in the brain (Figure 3) can damage the structure of biological macromolecules, causing synaptic dysfunction (downregulation of SNAP-25) and necrosis (Figure 2), as well as severe neuroinflammation (Figures 4–6). In the process of neuroinflammation, inappropriately activated microglia and reactive astrocytes can engulf synaptic material and suppress synaptic plasticity [7]. Furthermore, excessive release of pro-inflammatory cytokines (such as IL-1*β*, IL-6, and TNF-*α*) from microglia and astrocytes can exacerbate synaptic impairments, neuronal death, and inhibition of neurogenesis in the hippocampus and cortex [7]. As a result, AA-induced oxidative stress and neuroinflammation disrupted the expressions of BDNF by blocking ERK/CREB signaling in the brain (Figure 7), thereby inhibiting the neuroprotective effects of BDNF. Interestingly, we found for the first time that AA exposure increased the expression levels of A*β*1-42 and 1-40 in rat hippocampus and cortex (Figure 7), as the most abundant forms of A*β* in the brain. On the one hand, A*β* accumulation was considered to be caused by oxidative stress and pro-inflammatory factors after AA exposure, which further led to both neuronal death and downregulation of synaptic protein in the hippocampus and association cortices of rats [24, 25]. On the other hand, A*β* deposits would aggravate oxidative stress, neuroinflammation, and loss of BDNF expressions, forming a vicious circle [24, 25, 30]. Fortunately, we found that BAE supplementation effectively attenuated AA-induced oxidative stress and neuroinflammation, while improving ERK/CREB/BDNF signaling and A*β* deposition, which eventually contributed to the protection on neurons and synapses in rat brain against AA toxicity.

In our work, BAE administration significantly reduced AA-induced lipid peroxidation by decreasing MDA levels, and increased GSH levels as well as the expressions of several antioxidant enzymes (SOD, GSH-Px, and CAT) in the brain and serum (Figure 3), indicating its protective effects against oxidative stress in AA-treated rats. Consistently, our previous studies have demonstrated that BAE can also prevent mitochondrial ROS formation in the liver of mouse models after AA exposure [23]. Due to their hydrogen donating and metal chelating properties, anthocyanins exhibit efficient antioxidant and antiradical effects directly by functioning as free radical scavengers, chain breakers, metal complexing agents, and singlet oxygen quenchers [31]. In addition, anthocyanins are also considered to be activators of the transcription factor nuclear factor erythroid 2-related factor 2 (Nrf2), which plays a key role in modulating the expressions of cytoprotective antioxidant enzymes including SOD, GSH-Px, and CAT [32]. A previous study suggested that dietary supplementation of anthocyanins from black beans can prevent ROS and MDA levels and improve GSH expressions via stimulating the antioxidant system of Nrf2/heme oxygenase-1 (HO-1) pathways in the hippocampus of AD mice [32]. This means Nrf2 signaling may be linked to the neuroprotective function of BAE, which will be investigated in our further studies. Collectively, we proposed that oxidative stress can be an important intervention target of BAE against AA neurotoxicity.

Many antioxidants have exhibited favorable anti-inflammatory activities [11, 14]. Our results suggested that BAE significantly mitigated AA-induced microgliosis and astrocytosis (Figures 4 and 5), and rescued the increase of IL-1*β*, IL-6, and TNF-*α* in the hippocampus and cortex of rats (Figure 6). The remarkable anti-inflammatory effects of BAE may be due to the upregulation of GSH, which can suppress the activation of the inflammatory response-regulated transcription factor nuclear factor-*κ*B (NF-*κ*B), thus inhibiting glial cell reactivity and overproduction of pro-inflammatory cytokines [33]. Correspondingly, anthocyanins can also prevent microglia- and astrocytes-mediated neuroinflammation by suppressing NF-*κ*B activation in lipopolysaccharide (LPS)- and d-galactose-treated rodents [18, 19]. However, a previous study showed that bilberry anthocyanins activated the microglia via the CD33/triggering receptor expressed on myeloid cells 2 (TREM2)/tyro protein tyrosine kinase binding protein (TYROBP) signaling pathway, inducing phagocytosis of microglia to A*β* plaques in APP/PSEN1 transgenic mice [20]. These results in different animal models suggested that anthocyanins consumption may modulate the response of microglia to diverse environmental stimulus, through modifying the balance between different phenotypes of microglia (including M1 and M2 phenotypes) in the brain [20, 34].

BDNF is an important neurotrophic factor for synaptic plasticity and neurogenesis, highly expressed in the hippocampus and cortex [10]. It has been studied that elevated BDNF can mitigate the neuronal and synaptic injury in AA-exposed human neuroblastoma (NB-1) cells [35]. In this study, BAE significantly ameliorated the ERK/CREB/BDNF signaling suppressed by AA in the hippocampus (Figure 7), suggesting that the BDNF pathway may be a potential molecular target for dietary anthocyanin. Consistent with our findings, supplementation of pomegranate-derived anthocyanins can improve the CREB/BDNF pathways and alleviate cognitive impairments in an opioid-dependent rat model [36]. As previously reported, GSH deficiency and IL-1*β* administration can impair the expressions of BDNF in the hippocampus of rats [37, 38]. However, BNDF expressions can be promoted by free radical scavenging molecules (such as vitamin E [39], bikaverin [40], rosmarinic acid [41], and edaravone [42]) and anti-inflammatory ingredients (such as doxycycline [43] and minocycline [44]). Especially, many plant-derived polyphenols with strong antioxidant and anti-inflammatory abilities, including quercetin [45], resveratrol [46], apigenin [47], and naringenin [48], can increase BDNF levels in several animal models of neurological disorders. Based on these results, the upregulation of the BDNF pathway by BAE may be largely attributed to its resistance to oxidative stress and neuroinflammation, whereas it has been demonstrated that elevated BDNF levels can also stimulate Nrf2 activation through ERK phosphorylation, thereby inducing the transcription of downstream cytoprotective genes and reducing oxidative stress and related inflammatory response [49]. Therefore, BAE-upregulated BDNF may exert a positive feedback regulation on attenuating AA-induced oxidative stress and neuroinflammation, and further contribute to the protection of BAE against AA neurotoxicity, which will be confirmed in our follow-up experiments.

A*β* generation has been demonstrated to be induced by oxidative stress and neuroinflammation, and can result in increased synaptic dysfunction and neurodegeneration [25]. A*β* is released after sequential cleavage of amyloid precursor protein (APP) by *β*- and *γ*-secretases in the amyloidogenic pathway [25]. In our study, BAE dramatically reduced the levels of A*β*1-42 and 1-40 induced by AA in the hippocampus and cortex of rats (Figure 7). Consistently, anthocyanins have exhibited anti-A*β* aggregation effects in thermal- and methylglyoxal-induced fibrillation models [50], as well as cellular and animal models of AD [51]. There is evidence that polyphenols can form a ternary complex with A*β* peptides and metal ions via their metal chelating activity and hydrophobicity, thereby reducing the toxicity of metal-A*β* species and the amyloid fibril deposition in the brain [52]. In addition, because of the inhibitory effects on microglial activation, polyphenols may restrain the release of microglia-derived ASC specks, further decreasing the A*β* oligomer formation and aggression [53, 54]. Moreover, a previous study reported that though inhibiting the ROS production and microglial activation, anthocyanins from *Lycium ruthenicum* Murr can reduce *β*-site amyloid precursor protein cleaving enzyme 1 (BACE1) expressions and A*β* in d-galactose-treated rats [19]. These results indicated that the anti-amyloidogenic effect of BAE may be ascribed to its antioxidant and anti-inflammatory functions in AA-treated rats. Conversely, A*β* reduction would in turn trigger the inhibition of ROS and pro-inflammatory cytokine production as well as CREB/BDNF pathway activation in the brain, thereby promoting a virtuous cycle of neuroprotection [24, 25, 30].

Due to its vigorous antioxidant and anti-inflammatory effects, BAE alleviated AA-induced neuronal degeneration and downregulation of synaptic protein (SNAP-25) in rat hippocampus and cortex in the present study (Figure 2), which suggested BAE's promising effects on protecting neurons and synaptic functions. As an important presynaptic protein, SNAP-25 is involved in synaptic vesicle exocytosis and the regulation of voltage-gated calcium channels during the synaptic transmission, while the downregulation of SNAP-25 in the brain impairs the neurotransmitter release and synaptic potentiation [55]. In agreement with our findings, anthocyanins have been shown to improve the expressions of SNAP-25 and other synaptic-related proteins (such as SNAP-23, synaptophysin, and syntaxin) in the brain of LPS- and d-galactose-treated animal models [56, 57]. With the dynamic neuroprotective effects on neurons and synapses, anthocyanins exhibit the potential preventive and therapeutic properties not only for a few toxic factors including hydrogen peroxide, ethanol, acrolein, glutamate, and scopolamine, but also for a wide range of brain disorders, such as AD, Parkinson's disease, and cerebral ischemia [28]. Furthermore, the preparation and application of several anthocyanins-loaded nanoparticles would be beneficial to reinforce the neuroprotection of anthocyanins against A*β* by increasing the bioavailability and stability of anthocyanins, showing the therapeutic potential of anthocyanins in reducing AD pathology [58, 59].

## 5. Conclusions

**Figure 8:**
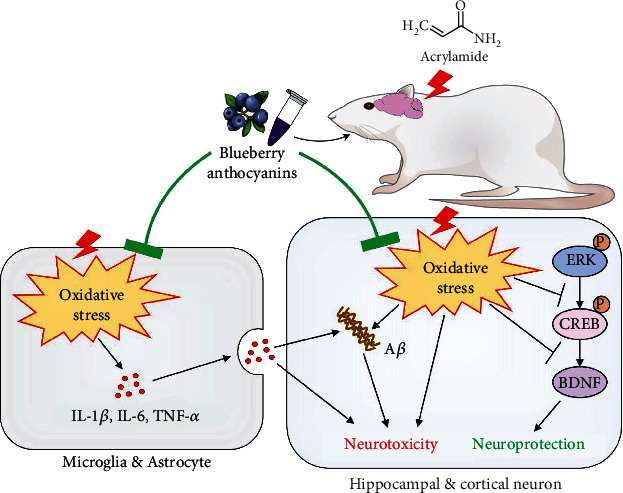
Schematic diagram of the neuroprotective effects of blueberry anthocyanins extract (BAE) against acrylamide (AA) exposure in rat hippocampus and cortex. BAE supplementation attenuated AA-induced oxidative stress and neuroinflammation, while improving the extracellular signal-related kinase (ERK)/cAMP response elements binding protein (CREB)/brain-derived neurotrophic factor (BDNF) pathway and relieving the accumulation of amyloid beta (A*β*), which eventually contributed to the neuroprotection against AA toxicity. IL-1*β*: interleukin-1*β*; IL-6: interleukin-6; TNF-*α*: tumor necrosis factor-alpha; P: phosphorylation.

In summary, BAE supplementation can effectively alleviate AA-induced neuronal and synaptic impairments. As proposed in Figure 8, our finding indicated that BAE attenuated the oxidative damage after AA exposure, resulting in reducing the neuroinflammation, elevating ERK/CREB/BDNF signaling expressions, and inhibiting A*β* deposition in the hippocampus and cortex of rats. Meanwhile, these beneficial effects may interact to create a positive feedback loop, thereby preventing AA neurotoxicity in neurons and synapses. The present study highlights the neuroprotection of BAE against AA exposure, and provides an effective intervention strategy for neurotoxins and neurological diseases through offering anthocyanins enriched foods.

## Data Availability

The data used to support the findings of this study are available from the corresponding author upon request.

## References

[1] Matoso V., Bargi-Souza P., Ivanski F., Romano M. A., Romano R. M. (2019). Acrylamide: a review about its toxic effects in the light of Developmental Origin of Health and Disease (DOHaD) concept. *Food Chemistry*.

[2] Erdemli Z., Erdemli M. E., Turkoz Y., Gul M., Yigitcan B., Gozukara Bag H. (2019). The effects of acrylamide and Vitamin E administration during pregnancy on adult rats testis. *Andrologia*.

[3] Erdemli M. E., Turkoz Y., Altinoz E., Elibol E., Dogan Z. (2016). Investigation of the effects of acrylamide applied during pregnancy on fetal brain development in rats and protective role of the vitamin E. *Human & Experimental Toxicology*.

[4] Liu Z. M., Tse L. A., Chen B. (2017). Dietary acrylamide exposure was associated with mild cognition decline among non-smoking Chinese elderly men. *Scientific Reports*.

[5] Erkekoglu P., Baydar T. (2014). Acrylamide neurotoxicity. *Nutritional Neuroscience*.

[6] Kopanska M., Muchacka R., Czech J., Batoryna M., Formicki G. (2018). Acrylamide toxicity and cholinergic nervous system. *Journal of Physiology and Pharmacology*.

[7] Zong C., Hasegawa R., Urushitani M. (2019). Role of microglial activation and neuroinflammation in neurotoxicity of acrylamide in vivo and in vitro. *Archives of Toxicology*.

[8] Zhao M., Wang F. S. L., Hu X. S., Chen F., Chan H. M. (2017). Effect of acrylamide-induced neurotoxicity in a primary astrocytes/microglial co-culture model. *Toxicology In Vitro*.

[9] Zhao M., Lewis Wang F. S., Hu X., Chen F., Chan H. M. (2017). Acrylamide-induced neurotoxicity in primary astrocytes and microglia: roles of the Nrf2-ARE and NF-*κ*B pathways. *Food and Chemical Toxicology*.

[10] Lima Giacobbo B., Doorduin J., Klein H. C., Dierckx R. A. J. O., Bromberg E., de Vries E. F. J. (2019). Brain-derived neurotrophic factor in brain disorders: focus on neuroinflammation. *Molecular Neurobiology*.

[11] Yan D., Yao J., Liu Y. (2018). Tau hyperphosphorylation and P-CREB reduction are involved in acrylamide- induced spatial memory impairment: suppression by curcumin. *Brain, Behavior, and Immunity*.

[12] Erdemli M. E., Arif Aladag M., Altinoz E. (2018). Acrylamide applied during pregnancy causes the neurotoxic effect by lowering BDNF levels in the fetal brain. *Neurotoxicology and Teratology*.

[13] Foroutanfar A., Mehri S., Kamyar M., Tandisehpanah Z., Hosseinzadeh H. (2020). Protective effect of punicalagin, the main polyphenol compound of pomegranate, against acrylamide-induced neurotoxicity and hepatotoxicity in rats. *Phytotherapy Research*.

[14] Goudarzi M., Mombeini M. A., Fatemi I. (2019). Neuroprotective effects of Ellagic acid against acrylamide-induced neurotoxicity in rats. *Neurological Research*.

[15] Motamedshariaty V. S., Amel Farzad S., Nassiri-Asl M., Hosseinzadeh H. (2014). Effects of rutin on acrylamide-induced neurotoxicity. *Daru*.

[16] Li D., Wang P., Luo Y., Zhao M., Chen F. (2017). Health benefits of anthocyanins and molecular mechanisms: update from recent decade. *Critical Reviews in Food Science and Nutrition*.

[17] Society C. N. (2013). *Chinese DRIs Handbook*.

[18] Khan M. S., Ali T., Kim M. W. (2016). Anthocyanins protect against LPS-induced oxidative stress-mediated neuroinflammation and neurodegeneration in the adult mouse cortex. *Neurochemistry International*.

[19] Chen S., Zhou H., Zhang G. (2019). Anthocyanins from Lycium ruthenicum Murr. ameliorated d-galactose-induced memory impairment, oxidative stress, and neuroinflammation in adult rats. *Journal of Agricultural and Food Chemistry*.

[20] Li J., Zhao R., Jiang Y. (2020). Bilberry anthocyanins improve neuroinflammation and cognitive dysfunction in APP/PSEN1 mice via the CD33/TREM2/TYROBP signaling pathway in microglia. *Food & Function*.

[21] Zhao M., Wang P., Zhu Y., Liu X., Hu X., Chen F. (2015). Blueberry anthocyanins extract inhibits acrylamide-induced diverse toxicity in mice by preventing oxidative stress and cytochrome P450 2E1 activation. *Journal of Functional Foods*.

[22] Wang P., Ji R., Ji J., Chen F. (2019). Changes of metabolites of acrylamide and glycidamide in acrylamide-exposed rats pretreated with blueberry anthocyanins extract. *Food Chemistry*.

[23] Zhao M., Wang P., Zhu Y., Liu X., Hu X., Chen F. (2015). The chemoprotection of a blueberry anthocyanin extract against the acrylamide-induced oxidative stress in mitochondria: unequivocal evidence in mice liver. *Food & Function*.

[24] Leng F., Edison P. (2021). Neuroinflammation and microglial activation in Alzheimer disease: where do we go from here?. *Nature Reviews. Neurology*.

[25] Dong H., Csernansky J. G. (2009). Effects of stress and stress hormones on amyloid-*β* protein and plaque deposition. *Journal of Alzheimer's Disease*.

[26] Li Y., Ma R., Xu Z. (2013). Identification and quantification of anthocyanins in Kyoho grape juice-making pomace, Cabernet Sauvignon grape winemaking pomace and their fresh skin. *Journal of the Science of Food and Agriculture*.

[27] Sánchez J., Cabrer J. M., Rosselló C. A., Palou A., Picó C. (2008). Formation of hemoglobin adducts of acrylamide after its ingestion in rats is dependent on age and sex. *Journal of Agricultural and Food Chemistry*.

[28] Zhang J., Wu J., Liu F. (2019). Neuroprotective effects of anthocyanins and its major component cyanidin-3-O-glucoside (C3G) in the central nervous system: an outlined review. *European Journal of Pharmacology*.

[29] Muldoon L. L., Alvarez J. I., Begley D. J. (2013). Immunologic privilege in the central nervous system and the blood-brain barrier. *Journal of Cerebral Blood Flow and Metabolism*.

[30] Amidfar M., de Oliveira J., Kucharska E., Budni J., Kim Y. K. (2020). The role of CREB and BDNF in neurobiology and treatment of Alzheimer's disease. *Life Sciences*.

[31] Prior R. L., Wu X. (2006). Anthocyanins: structural characteristics that result in unique metabolic patterns and biological activities. *Free Radical Research*.

[32] Ali T., Kim T., Rehman S. U. (2018). Natural dietary supplementation of anthocyanins via PI3K/Akt/Nrf2/HO-1 pathways mitigate oxidative stress, neurodegeneration, and memory impairment in a mouse model of Alzheimer's disease. *Molecular Neurobiology*.

[33] Bjørklund G., Peana M., Maes M., Dadar M., Severin B. (2021). The glutathione system in Parkinson's disease and its progression. *Neuroscience and Biobehavioral Reviews*.

[34] Meireles M., Marques C., Norberto S. (2016). Anthocyanin effects on microglia M1/M2 phenotype: consequence on neuronal fractalkine expression. *Behavioural Brain Research*.

[35] Chen X., Xiao J. W., Cao P. (2021). Brain-derived neurotrophic factor protects against acrylamide-induced neuronal and synaptic injury via the TrkB-MAPK-Erk1/2 pathway. *Neural Regeneration Research*.

[36] Ridzwan N., Jumli M. N., Baig A. A., Rohin M. A. K. (2020). Pomegranate-derived anthocyanin regulates MORs-cAMP/CREB-BDNF pathways in opioid-dependent models and improves cognitive impairments. *Journal of Ayurveda and integrative medicine*.

[37] Barrientos R. M., Sprunger D. B., Campeau S., Watkins L. R., Rudy J. W., Maier S. F. (2004). BDNF mRNA expression in rat hippocampus following contextual learning is blocked by intrahippocampal IL-1*β* administration. *Journal of Neuroimmunology*.

[38] Lech M. A., Leśkiewicz M., Kamińska K., Rogóż Z., Lorenc-Koci E. (2021). Glutathione deficiency during early postnatal development causes schizophrenia-like symptoms and a reduction in BDNF levels in the cortex and hippocampus of adult Sprague-Dawley rats. *Int J Mol Sci*.

[39] Erdemli Z., Erdemli M. E., Turkoz Y. (2021). Vitamin E effects on developmental disorders in fetuses and cognitive dysfunction in adults following acrylamide treatment during pregnancy. *Biotechnic & Histochemistry*.

[40] Nirmaladevi D., Venkataramana M., Chandranayaka S., Ramesha A., Jameel N. M., Srinivas C. (2014). Neuroprotective effects of bikaverin on H2O2-induced oxidative stress mediated neuronal damage in SH-SY5Y cell line. *Cellular and Molecular Neurobiology*.

[41] Ghaffari H., Venkataramana M., Jalali Ghassam B. (2014). Rosmarinic acid mediated neuroprotective effects against H_2_O_2_-induced neuronal cell damage in N2A cells. *Life Sciences*.

[42] Wu H. T., Yu Y., Li X. X. (2021). Edaravone attenuates H_2_O_2_ or glutamate-induced toxicity in hippocampal neurons and improves AlCl_3_/D-galactose induced cognitive impairment in mice. *Neurotoxicology*.

[43] Mello B. S., Monte A. S., McIntyre R. S. (2013). Effects of doxycycline on depressive-like behavior in mice after lipopolysaccharide (LPS) administration. *Journal of Psychiatric Research*.

[44] Zhang C., Zhang Y. P., Li Y. Y. (2019). Minocycline ameliorates depressive behaviors and neuro-immune dysfunction induced by chronic unpredictable mild stress in the rat. *Behavioural Brain Research*.

[45] Fang K., Li H. R., Chen X. X. (2020). Quercetin alleviates LPS-induced depression-like behavior in rats via regulating BDNF-related imbalance of Copine 6 and TREM1/2 in the hippocampus and PFC. *Frontiers in Pharmacology*.

[46] Ge L., Liu L., Liu H. (2015). Resveratrol abrogates lipopolysaccharide-induced depressive-like behavior, neuroinflammatory response, and CREB/BDNF signaling in mice. *European Journal of Pharmacology*.

[47] Sharma P., Sharma S., Singh D. (2020). Apigenin reverses behavioural impairments and cognitive decline in kindled mice via CREB-BDNF upregulation in the hippocampus. *Nutritional Neuroscience*.

[48] Olugbemide A. S., Ben-Azu B., Bakre A. G., Ajayi A. M., Femi-Akinlosotu O., Umukoro S. (2021). Naringenin improves depressive- and anxiety-like behaviors in mice exposed to repeated hypoxic stress through modulation of oxido-inflammatory mediators and NF-kB/BDNF expressions. *Brain Research Bulletin*.

[49] Santana-Martínez R. A., Silva-Islas C. A., Fernández-Orihuela Y. Y. (2019). The therapeutic effect of curcumin in quinolinic acid-induced neurotoxicity in rats is associated with BDNF, ERK1/2, Nrf2, and antioxidant enzymes. *Antioxidants*.

[50] Ma H., Johnson S. L., Liu W. (2018). Evaluation of polyphenol anthocyanin-enriched extracts of blackberry, black raspberry, blueberry, cranberry, red raspberry, and strawberry for free radical scavenging, reactive carbonyl species trapping, anti-glycation, anti-*β*-amyloid aggregation, and microglial neuroprotective effects. *International Journal of Molecular Sciences*.

[51] Yamakawa M. Y., Uchino K., Watanabe Y. (2016). Anthocyanin suppresses the toxicity of A*β* deposits through diversion of molecular forms in in vitro and in vivo models of Alzheimer's disease. *Nutritional Neuroscience*.

[52] Lakey-Beitia J., Burillo A. M., La Penna G., Hegde M. L., Rao K. S. (2021). Polyphenols as potential metal chelation compounds against Alzheimer's disease. *Journal of Alzheimer's Disease*.

[53] Hussain T., Tan B., Yin Y., Blachier F., Tossou M. C., Rahu N. (2016). Oxidative stress and inflammation: what polyphenols can do for us?. *Oxidative Medicine and Cellular Longevity*.

[54] Venegas C., Kumar S., Franklin B. S. (2017). Microglia-derived ASC specks cross-seed amyloid-*β* in Alzheimer's disease. *Nature*.

[55] Antonucci F., Corradini I., Fossati G., Tomasoni R., Menna E., Matteoli M. (2016). SNAP-25, a known presynaptic protein with emerging postsynaptic functions. *Frontiers in synaptic neuroscience*.

[56] Khan M. S., Ali T., Kim M. W., Jo M. H., Chung J. I., Kim M. O. (2019). Anthocyanins improve hippocampus-dependent memory function and prevent neurodegeneration via JNK/Akt/GSK3*β* signaling in LPS-treated adult mice. *Molecular Neurobiology*.

[57] Rehman S. U., Shah S. A., Ali T., Chung J. I., Kim M. O. (2017). Anthocyanins reversed D-galactose-induced oxidative stress and neuroinflammation mediated cognitive impairment in adult rats. *Molecular Neurobiology*.

[58] Amin F. U., Shah S. A., Badshah H., Khan M., Kim M. O. (2017). Anthocyanins encapsulated by PLGA@PEG nanoparticles potentially improved its free radical scavenging capabilities via p38/JNK pathway against A*β*1–42-induced oxidative stress. *Journal of nanobiotechnology*.

[59] Kim M. J., Rehman S. U., Amin F. U., Kim M. O. (2017). Enhanced neuroprotection of anthocyanin-loaded PEG-gold nanoparticles against A*β*_1-42_-induced neuroinflammation and neurodegeneration _via_ the NF-_K_B /JNK/GSK3 *β* signaling pathway. *Nanomedicine*.

